# Variations in Plant Water Use Efficiency Response to Manipulated Precipitation in a Temperate Grassland

**DOI:** 10.3389/fpls.2022.881282

**Published:** 2022-05-19

**Authors:** Xuying Hai, Jianping Li, Jiwei Li, Yulin Liu, Lingbo Dong, Xiaozhen Wang, Wenwen Lv, Zhenhong Hu, Zhouping Shangguan, Lei Deng

**Affiliations:** ^1^State Key Laboratory of Soil Erosion and Dryland Farming on the Loess Plateau, Northwest A&F University, Yangling, China; ^2^School of Agriculture, Ningxia University, Yinchuan, China; ^3^Institute of Soil and Water Conservation, Chinese Academy of Sciences and Ministry of Water Resources, Yangling, China

**Keywords:** climate change, grassland, precipitation, soil microbe, soil nutrients, soil temperature, water use efficiency

## Abstract

Water use efficiency (WUE) plays important role in understanding the interaction between carbon and water cycles in the plant-soil-atmosphere system. However, little is known regarding the impact of altered precipitation on plant WUE in arid and semi-arid regions. The study examined the effects of altered precipitation [i.e., ambient precipitation (100% of natural precipitation), decreased precipitation (DP, −50%) and increased precipitation (IP, +50%)] on the WUE of grass species (*Stipa grandis* and *Stipa bungeana*) and forb species (*Artemisia gmelinii*) in a temperate grassland. The results found that WUE was significantly affected by growth stages, precipitation and plant species. DP increased the WUE of *S. grandis* and *S. bungeana* generally, but IP decreased WUE especially in *A. gmelinii*. And the grasses had the higher WUE than forbs. For different growth stages, the WUE in the initial growth stage was lower than that in the middle and late growth stages. Soil temperature, available nutrients (i.e., NO_3_^–^, NH_4_^+^, and AP) and microorganisms under the altered precipitations were the main factors affecting plant WUE. These findings highlighted that the grasses have higher WUE than forbs, which can be given priority to vegetation restoration in arid and semi-arid areas.

## Introduction

Precipitation patterns are important environmental factors that affect the structure and process of terrestrial ecosystems and the key drivers of biological activity in semi-arid and arid ecosystems (Nielsen and Ball, 2015). With the intensification of climate change, the global precipitation pattern has undergone enormous changes, and the frequency of extreme manipulation events has been increasing (Sponseller, 2010). This affects the structure and function of plants and microbial communities, which in turn influences the water, energy and nutrient cycles of terrestrial ecosystems (Weltzin et al., 2003).

Water use efficiency (WUE) is the ratio of carbon (C) assimilation to water losses, which reflects the interaction between the C and water cycles for the plant-soil-atmosphere system (Bai et al., 2020). Moreover, it is also an important indicator to explore the adaptability of plants to environmental change and to predict the impact of global change (Nielsen and Ball, 2015). At the leaf scale, WUE is the ratio of photosynthetic rate to transpiration rate (Bai et al., 2020). C isotope fractionation is highly correlated with the ratio of photosynthetic C assimilation and transpiration rate, and C isotope fractionation is also highly correlated with plant WUE (Latif et al., 2014). Stable C isotope technology is used globally to determine WUE, and the main principle of this technology is that there is a strong correlation between the stable C isotope ratio (^13^C/^12^C, δ^13^C) or stability C isotope discrimination coefficient (Δ) and WUE of C3 plants, thus can be used it as an indicator of WUE (Latif et al., 2014). It is an effective method to study the long-term WUE of plants and reflects the use of water and adaptation of plants to water stress over time (Chen et al., 2011).

The factors affecting plant WUE mainly including plant species, environmental and regional factors, especially, soil moisture (SM) is one of the most direct factors (Law et al., 2010). Under limited soil water conditions, plants can control water losses and increase their WUE by reducing stomatal conductance, which varies among plant species and depends on water availability and carbon dioxide concentration in the atmosphere (Latif et al., 2014; Silva, 2015). Precipitation deficiency is frequently recorded as a result of global warming, and affects the terrestrial C and water cycles by reducing the C sequestration ability and aggravating the evaporation rate of plants (Battipaglia et al., 2014), which further influences the WUE of plants. However, one study reported that plant WUE also decrease with drought in semi-arid regions (Yang et al., 2016). Moreover, previous studies paid more attentions on the precipitation changes effect on WUE at the community scale, little information at the plant species scale. Furthermore, plant species is the key factor determined the WUE under the changes in precipitation patterns. Therefore, it is necessary to explore the variation tendency in plant WUE at the species scale, which are crucial for predicting the impact of future climatic change on plant C and water cycling processes.

The grassland area of the world is approximately 50 million km^2^, accounting for approximately 33.5% of global land area (Kang et al., 2007). As an important subsystem of terrestrial ecosystems, grasslands play a key role in global change and ecosystem function. Considering the background of global climate change, alterations in precipitation patterns will affect water utilization in plants directly and the functions and processes of grassland ecosystems indirectly. However, most of the previous studies had only focused on short-term (at the day scale) WUE of plants, a single plant species or single growth period responded to altered precipitation (Karatassiou and Noitsakis, 2010; Sensuła, 2015; Han et al., 2020). Therefore, this study first to explore the effect of altered precipitation on WUE among functional groups (two grasses and one forbs) following the whole growth period, and further to identify the key abiotic and biotic factors effecting on the dynamic of WUE in temperate grasslands. It would be useful to better understand the changes and the response mechanisms of WUE to altered precipitation patterns in grassland ecosystems.

## Materials and Methods

### Study Area

This study was conducted at the national fixed grassland monitoring point (106°17′49.2′′E, 36°16′44.5′′N, 1,963 m a.s.l.) in the Yunwu Mountains on the Loess Plateau of China, where has a temperate continental climate and is located inland. The mean annual temperature is 7.12°C. The mean annual precipitation of 439.3 mm and where has a strong rainfall seasonality (mainly occurring between June and September). The soil types were gray cinnamonic soil and loessial soil. The study area is a typical temperate grassland, and the main vegetation types are *Stipa. grandis*, *Stipa. bungeana*, *Artemisia. gmelinii*, *Agropyron cristatum*, and *Heteropappus altaicus et al*.

### Experimental Design and Sampling

**FIGURE 1:**
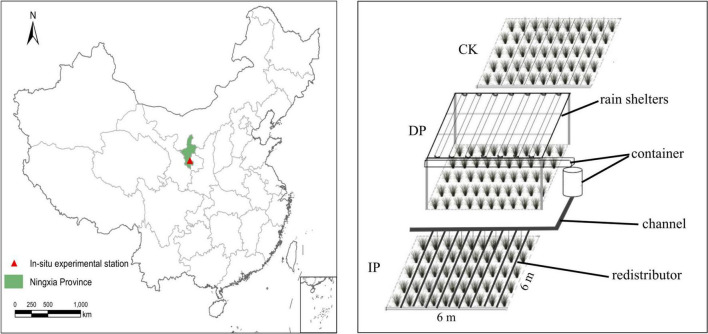
Schematic diagramof study area and experiment design.

The experiment platform was built in 2017 where as a randomized complete block design with five replicate blocks in the grassland. In each block, three 6 m × 6 m plots were established with a 1 m buffer zone between each plot. A transparent plastic rain sheltering rack (the uniformly laid rain shelter was constructed with PVC with a length of 5 m, a groove width of 30 cm, and a light transmittance of 95%) was adopted to support the collecting bucket and the artifact drip irrigation system to realize real-time collection and redistribution of the natural precipitation in the plots. Rain shelters intercepted half of the ambient precipitation (control) to form a reduced precipitation treatment (DP). The intercepted water was piped to an adjacent plot to form an increased precipitation manipulated (IP). To prevent the interference of soil moisture from the outside, 1-m deep PVC boards were embedded around the perimeter to prevent the surface soil moisture from seeping sideways (Figure 1).

The terminology of Allen et al. (2011) was used as the basis to divide all the plants into the two functional groups: grass (plant species of the *Poaceae* family) and forb (herbaceous, dicotyledonous broad-leaved plant). Based on a field survey, we found that there had three dominant species that are *S. grandis*, *S. bungeana*, and *A. gmelinii*, which were typical zonal vegetation and the main constructive species in the hilly and gully region on Loess Plateau. The three plants can be divided into two functional groups: grass species (*S. grandis* and *S. bungeana*) and forb species (*A. gmelinii*).

During the initial (May), middle (July), and final (September) stages of the growing season of 2019, we harvested the aboveground biomass of individuals in these plots, the biomass was mixed to form a composite sample, stored in ice boxes, and brought back to the laboratory to measure relevant indexes. The plant δ^13^C were analyzed by isotope ratio mass spectrometer (IRMS, Thermo-Fisher Scientific, Bremen, Germany) after the samples were dried at 60°C for 72 h. After removing the litter layer, soil samples were collected from each quadrat. In each plot, three soil cores (5 cm diameter) were randomly collected with an “S” shape using a soil drilling sampler (9 cm inner diameter), and the soils were combined to provide one composite soil sample per plot. The soil samples were passed through a 2 mm sieve to remove roots and stones. Then, all soil samples were separated into two parts; one part was used for the determination of soil properties (air-dried and stored at 25°C) and the other part was utilized for soil microbial biomass (stored at 4°C).

### Measurement of the Abiotic and Biotic Properties

Soil moisture (%) and soil temperature (ST, °C) were measured using a soil moisture meter (Takeme-10, Mianyang, China). The soil organic carbon content (SOC) was analyzed using exothermic heating and oxidation using the potassium dichromate method (Nelson and Sommers, 1982). The soil total nitrogen content (TN) was determined using the Kjeldahl method (Bremner, 1996). The soil available nitrogen content (AN) was determined using the alkali-hydrolyzed diffusion method (Stanford, 1982). Soil total phosphorus (TP) and available phosphorus (AP) were determined using melted molybdenum, antimony, and scandium colorimetry (Olsen and Sommers, 1982). Soil nitrate nitrogen (NO_3_^–^–N) and ammoniacal nitrogen content (NH_4_^+^–N) were measured using a continuous flow analytical system (Autoanalyzer 3, Bran and Luebbe, Germany).

Microbial biomass for carbon (MBC), nitrogen (MBN), and phosphorus (MBP) concentrations were analyzed using the chloroform fumigation extraction method (Vance et al., 1987). The specific experimental methods referred to Li J. W. et al. (2019).

### Calculation of Carbon Isotope Discrimination and Water Use Efficiency

The isotopic discrimination in photosynthesis can be described by the model proposed by Farquhar et al. (1989):


(1) $${△}^{13}C\left(\text{‰}\right)=a+\left(b-a\right)C_{i}/C_{a}$$

Where *C*_*i*_ is the intercellular CO_2_ concentration, *C*_*a*_ is the ambient CO_2_ concentration, *a* (4.4‰) represents the fractionation effect that occurs during CO_2_ diffusion through the stomata, and *b* (27‰) represents the discrimination associated with carboxylation. The leaf conductivity used for water vapor loss (*g*_*H*2*O*_) and CO_2_ absorption (*g*_*CO*2_) has the following relationship:


(2) $$g_{H_{2}O}=1.6g_{co_{2}}g$$

In addition, the net photosynthetic rate (*A*) of the plant has the following relationship with *gCO*_2_ (Hietz et al., 2005):


(3) $$\text{A}=g_{H_{2}O}(C_{a}-C_{i)}$$

Combined with the above formula, WUE (*A*/*gH*_2_*O*) can be determined by Δ^13^C:


(4) $${△}^{13}C\left(‰\right)=a+\left(b-a\right)\left(1-\frac{1.6A}{C_{a}}g_{H_{2}O}\right)$$

So,


(5) $$\text{WUE}=\frac{\text{A}}{g_{H_{2}O}}=\left(b-{△}^{13}C\right)/1.6\left(b-a\right)$$

where *A* is the net photosynthesis, *g* is the stomatal conductance, and 1.6 is the ratio of diffusivities of water and CO_2_ in air.

### Statistical Analyses

Two-way ANOVA were performed to test the main effects of precipitation manipulated, growth stages, and their interactive effects on WUE, soil properties and ecological stoichiometry characteristics of the microbial biomass. Significance was evaluated at the 0.05 level (*P* < 0.05). We have carried out normality analysis and the test of homogeneity of variance before ANOVA analysis. When significance was observed at the *P* < 0.05 level, Post Hoc Duncan’s multiple range test was used to carry out the multiple comparisons. Pearson correlation analysis was used to determine the relationship between WUE and abiotic and biotic properties in the soil. Furthermore, a stepwise regression model was applied to detect the main factors affecting soil and microbial properties that affected plant WUE. The structural equation modeling framework was performed using Amos 22.0 to estimate the direct and indirect effects of environmental variables on plant WUE.

## Results

### Response of Plant Water Use Efficiency to Precipitation Manipulation at Different Growth Stages

**FIGURE 2:**
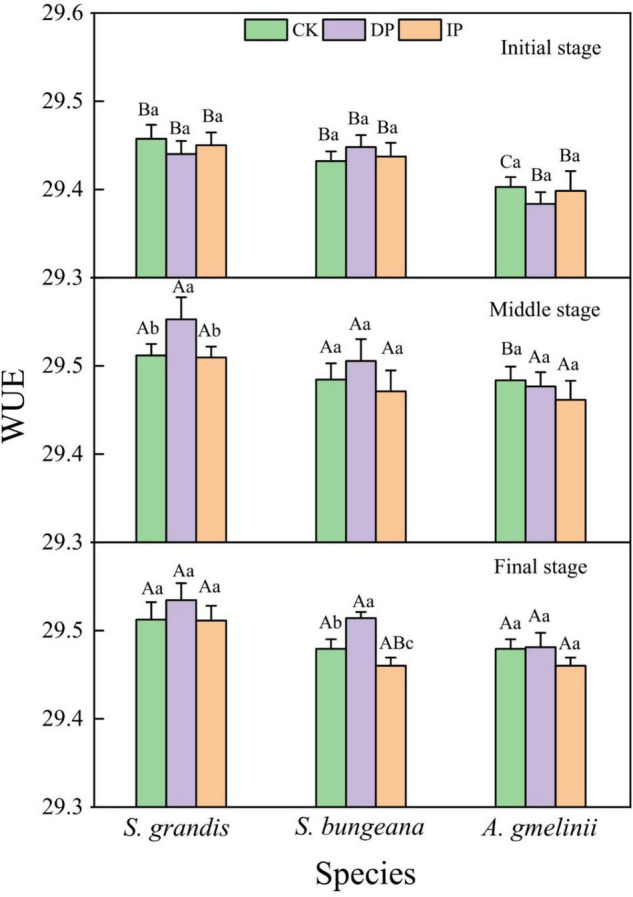
Response of plant WUE to precipitation manipulation at different growth stages. CK, ambient precipitation; DP, decrease precipitation (–50%); IP, increase precipitation (+50%); WUE, water use efficiency. Different small letters mean significant difference among different treatments at the 0.05 level; different capital letters mean significant difference among different growing stages at the 0.05 level.

Plant WUE was significantly affected by growth stage, precipitation treatment, and their interaction (*P* < 0.001; Table 1). For *S. grandis* and *S. bungeana*, the WUE of the DP was higher than ambient precipitation and IP generally, among them, significant differences were found in the middle growth stage of *S. grandis* and in the final growth stage of *S. bungeana*. However, IP decreased WUE, especially in *S. bungeana* and *A. gmelinii* (Figure 2). Among the different growth stages, the WUE of the *S. grandis*, *S. bungeana*, and *A. gmelinii* in the initial growth stage was lower than that in the other growth stages.

**TABLE 1:** ANOVA (*P*-value) analysis of the effects of growth stages and precipitation manipulation on plant WUE, soil properties, and microbial biomass ecological stoichiometry characteristics.

Factor	WUE	SM	ST	SOC	TN	TP	SOC:TN	SOC:TP	TN:TP	NH_4_^+^–N	NO_3_^–^–N	AHN	AP	MBC	MBN	MBP	MBC:MBN	MBC:MBP	MBN:MBP
Growing stages	0.00***	0.00***	0.00***	0.00***	0.03*	0.92	0.00***	0.00***	0.07	0.00***	0.00***	0.21	0.00***	0.00***	0.00***	0.38	0.13	0.00***	0.01*
Treatments	0.00***	0.00***	0.00***	0.23	0.10	0.52	0.63	0.23	0.08	0.05	0.00***	0.28	0.21	0.00***	0.26	0.03*	0.36	0.32	0.40
Growing stages × Treatments	0.00***	0.18	0.01*	0.98	0.66	0.42	0.41	0.72	0.38	0.28	0.00***	0.58	0.00***	0.53	0.00***	0.36	0.61	0.61	0.52

CK, ambient precipitation; DP, decrease precipitation (50%); IP, increase precipitation (50%); SM, soil moisture; ST, soil temperature; SOC, soil organic carbon; TN, soil total nitrogen; TP, soil total phosphorus; SOC:TN, SOC:TN ratio; SOC:TP, SOC:TP ratio; TN:TP, TN:TP ratio; NH_4_^+^–N, soil NH_4_^+^–N content; NO_3_^–^–N, soil NO_3_^–^–N content; AN, soil available nitrogen content; AP, soil available phosphorus content; MBC, microbial biomass carbon; MBN, microbial biomass nitrogen; MBP, microbial biomass phosphorus; MBC:MBN, MBC:MBN ratio; MBC:MBP, MBC:MBP ratio; MBN:MBP, MBN:MBP ratio. *P < 0.05; ***P < 0.001.

### Correlation Analysis of Factors Affecting Water Use Efficiency With Precipitation Manipulation

**FIGURE 3:**
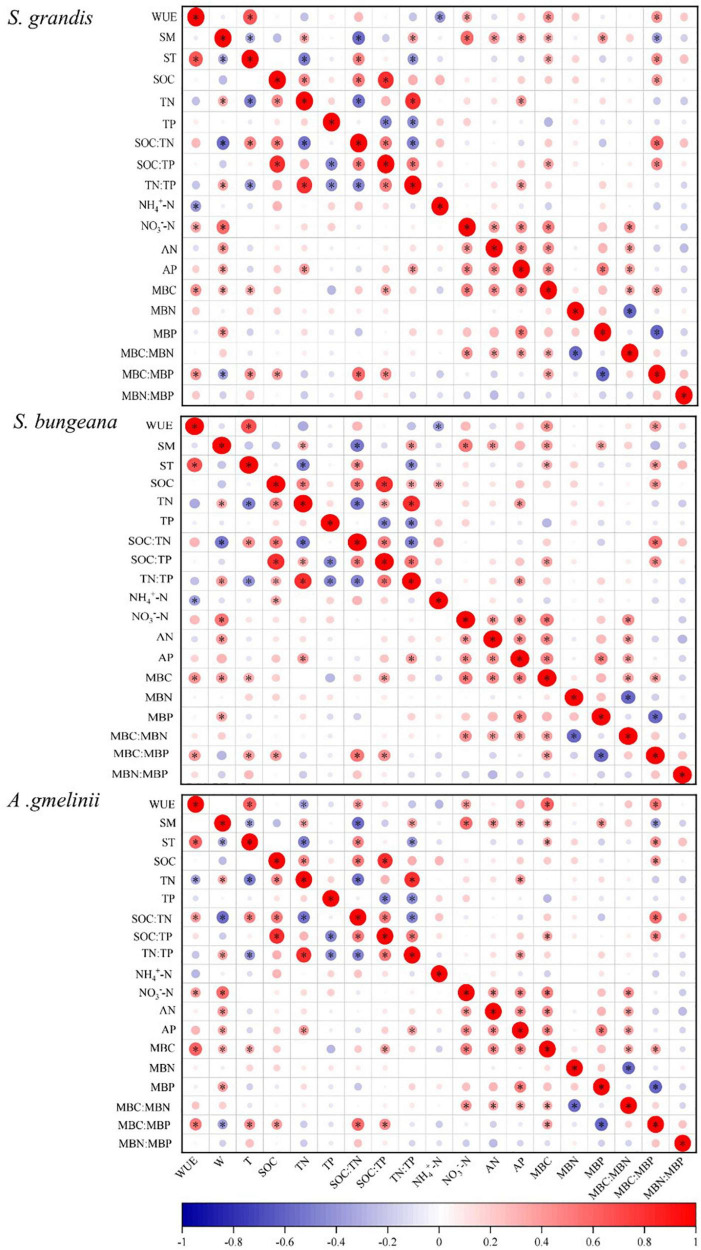
Correlation analysis of factors affecting WUE with precipitation manipulation. CK, ambient precipitation; DP, decrease precipitation (–50%); IP, increase precipitation (+50%). WUE, water use efficiency; SM, soil moisture; ST, soil temperature; SOC, soil organic carbon; TN, soil total nitrogen; TP, soil total phosphorus; SOC:TN, SOC:TN ratio; SOC:TP, SOC:TP ratio; TN:TP, TN:TP ratio; NH_4_^+^–N, soil NH_4_^+^–N content; NO_3_^–^–N, soil NO_3_^–^–N content; AN, soil available nitrogen content; AP, soil available phosphorus content; MBC, microbial biomass carbon; MBN, microbial biomass nitrogen; MBP, microbial biomass phosphorus; MBC:MBN, MBC:MBN ratio; MBC:MBP, MBC:MBP ratio; MBN:MBP, MBN:MBP ratio. Red indicates positive correlation and blue indicates negative correlation. The larger the circle, the greater the absolute value of the correlation coefficient. *P ≤ 0.05.

For *S. grandis*, the factors that were significantly related to WUE were ST, NH_4_^+^−N, NO_3_^–^–N, MBC, and MBC:MBP. For *S. bungeana*, ST, NH_4_^+^–N, MBC, and MBC:MBP were significantly correlated with WUE. The WUE of *A. gmelinii* was significantly correlated with ST, TN, SOC:TN, NO_3_^–^–N, MBC, and MBC:MBP (Figure 3). Moreover, the stepwise regression models detected that the WUE of *S. grandis* was mainly influenced by NO_3_^–^–N and NH_4_^+^–N, ST, NO_3_^–^–N, and NH_4_^+^–N had strong effects on WUE of *S. bungeana*, and the influencing factors for *A. gmelinii* were ST, MBC:MBP, NO_3_^–^–N, AP, and TN (Table 2).

**TABLE 2:** Multiple regression equation between WUE and soil properties and microbial biomass ecological stoichiometry characteristics.

Species	Multi-regression equation	*P*-value
*S. grandis*	y = 0.006 + 0.005*NO*_3_^–^*–N* − 0.007*NH_4_^+^–N*	0.00***
*S. bungeana*	y = 29.376 + 0.005*ST* − 0.005*NH_4_^+^–N* + 0.002*NO*_3_^–^*–N*	0.00***
*A. gmelinii*	y = 29.403+ 0.004*ST* + 0.001*MBC:MBP* + 0.004*NO*_3_^–^*–N* + 0.01*AP* − 0.07*TN*	0.00***

*ST, soil temperature; TN, soil total nitrogen; NH_4_^+^–N, soil NH_4_^+^–N content; NO_3_^–^–N, soil NO_3_^–^−N content; MBC:MBP, MBC:MBP ratio.*

***P < 0.001.

### Structural Equation Model of Soil Properties and Microbial Biomass Ecological Stoichiometry Characteristics Affecting Water Use Efficiency With Precipitation Manipulation

**FIGURE 4:**
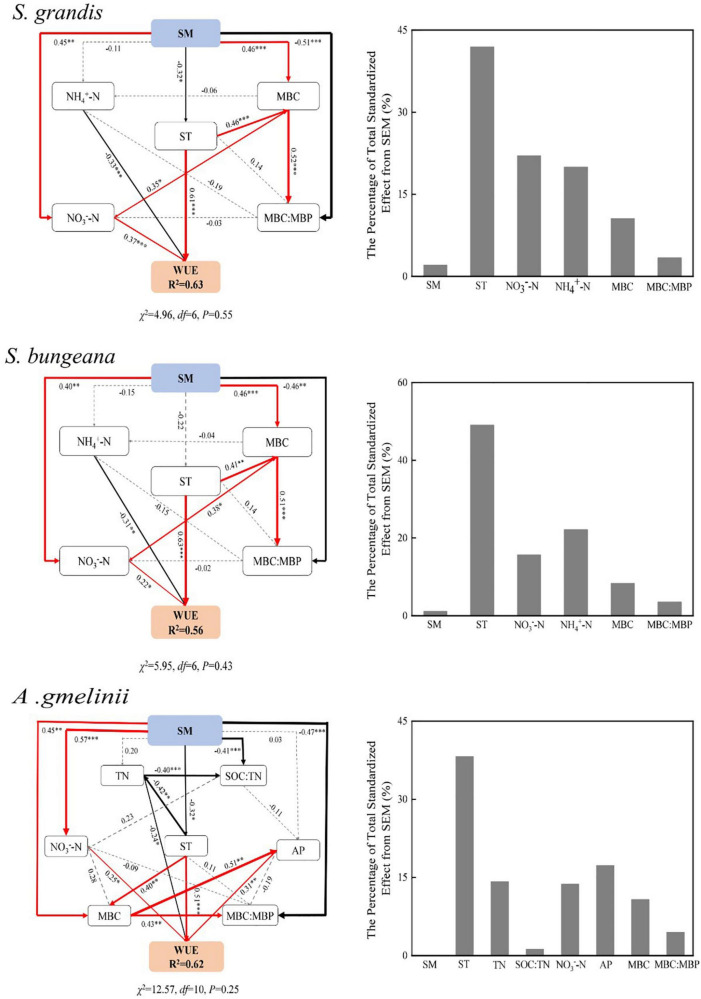
Structural equation model of the effect of soil properties and microbial biomass ecological stoichiometry on WUE with precipitation manipulation. SM, soil moisture; ST, soil temperature; TN, soil total nitrogen; SOC:TN, soil organic carbon: soil total nitrogen; NH_4_^+^–N, soil NH_4_^+^–N content; NO_3_^–^–N, soil NO_3_^–^–N content; AP, soil available phosphorus content; MBC, microbial biomass carbon; MBN, microbial biomass nitrogen; MBP, microbial biomass phosphorus; MBC:MBN, MBC:MBN ratio; MBC:MBP, microbial biomass carbon: microbial biomass phosphorus ratio. The final model fit the data well: *S. grandis* (χ^2^ = 4.96, *df* = 6, *P* = 0.55); *S. bungeana* (χ^2^ = 5.95, *df* = 6, *P* = 0.43); *A. gmelinii* (χ^2^ = 12.57, *df* = 10, *P* = 0.25). Numbers at arrows are standardized path coefficients. Width of the arrows indicates the strength of the relationships. The red lines indicate positive and significant, the black lines indicate negative significant, and the dashed lines indicate insignificant, respectively; R^2^ values indicate the proportion of the variance explained for each endogenous variable; result for goodness-of-fit tests are also reported underneath each plot (*P* > 0.05 indicates a good fit); *P < 0.05; **P < 0.01; ***P < 0.001.

The final structural equation model accurately fitted the data describing the interaction pathways among WUE, soil properties, and microbial biomass ecological stoichiometry in response to altered precipitation (Figure 4).

The main factors associated with the WUE of *S. grandis* and *S. bungeana* were NO_3_^–^–N, NH_4_^+^–N, ST, MBC, and MBC:MBP, and the final model explained 63 and 56% of the variation in WUE of *S. grandis* and *S. bungeana*, respectively. In addition, ST, TN, SOC:TN, AP, NO_3_^–^–N, MBC, and MBC:MBP played a critical role in the WUE of *A. gmelinii*, and the final model explained 62% of the variation in WUE. As a driving force, SM indirectly affects WUE by having positive or negative effects on other factors.

## Discussion

### Effects of Precipitation Manipulation on Plant Water Use Efficiency

Higher WUE indicates generally more dry matter production or C uptake per water loss (Gao et al., 2009). Generally, DP increased WUE and IP decreased WUE (Figure 2). Plants have higher WUE under drought, mainly because the stomatal conductance and transpiration rate of leaves decrease, but the change in net photosynthetic rate is relatively small (Latif et al., 2014). However, it is different in increasing photosynthesis and stomatal conductance of plants when higher SM under the IP conditions. The same increase in precipitation will greatly increase the amount of water available for evaporation, but which has little effect on promoting the photosynthetic rate of plants (Tian et al., 2010), thus reducing the WUE of plants.

Certain studies have reported that plants of different functional group are characterized by diversities in ecological strategies (Davison et al., 2020). Such variable strategies make plants to regulate the ability of resource absorption, utilization and transformation under changing environments, indicating mutable phenotypic plasticity of different function groups (Davison et al., 2020). The unique biological characteristics of grasses and forbs have adopted an underground strategy for storing in infertile soils potentially (Chapin, 1980). In our study, the WUE of *A. gmelinii* under different precipitation treatments showed no obvious change trend (Figure 2), and generally the WUE was higher in grasses than forbs given its larger leaf area. Although leaves are beneficial for intercepting the maximum light resources, it also means more water loss. Therefore, plant controls water loss through stomatal regulation resulting in a reduction in photosynthetic rate per unit area and thus leading to lower WUE, which showed a trade-off between different functional groups to maximize access to light and control water loss (Reich et al., 1997) and indicated that future vegetation restoration can be dominated by the functional groups with high resource competitiveness and WUE such as grasses in arid and semi-arid regions.

Plants WUE is synchronized with the total primary productivity, which is lower at the beginning and the end of the growing period, but higher at the growth peak (Tang et al., 2015), suggesting WUE varied with plant growth. However, our study showed that the WUE of the plants in the middle and final growth stages was significantly higher than that at the initial stage of growth (Figure 2). The reasons may be that in the middle and final growth stages when the temperature, amount of water evapotranspiration and the water demand of plants is high, the imbalance between water loss caused by vegetation evapotranspiration and the amount of SM replenishment will make the available water more and more short (Reynolds et al., 2004) and increase the plant WUE.

### Effects of Soil Hydrothermal Conditions on Plant Water Use Efficiency

The SM is the main determinant of plant WUE (Law et al., 2010). However, our study found that SM could not adequately explain the change in WUE (Figures 3, 4), indicating that small precipitation events do not effectively replenish the water available to plants in the arid area (Wright et al., 2001). At the same time, the weak correlation between WUE and soil water content may also be caused by the fact that the ecohydrological process mainly depends on the amount of water available in the phenological period, and the supplementation of SM through precipitation does not necessarily coincide with plant demand (Dong et al., 2011). In addition, precipitation has a delayed effect, which means that the effect of supplementation of SM on plants growth and community structure is a cumulative process (Sala et al., 2012; Bunting et al., 2017). Therefore, the effects of precipitation change on plant community may not be reflected if only did one growing season’s study. Therefore, long-term observation data are urgently needed to explore the effects of precipitation changes on the structure and function for plants.

Temperature is also an important factor affecting plant C isotope fractionation, which affect C isotope fractionation by regulating *C*_*i*_/*C*_*a*_ (Liu and Li, 2007), and affect plant WUE by causing a photosynthetic carboxylase reaction directly. Previous studies have found that WUE decreases with an increase in temperature due to high temperatures limit the stomatal conduction of plants (Ponton et al., 2006). With increasing temperature, the water vapor pressure difference between the inside and outside of the leaves increases and the transpiration of plants becomes intense, leading to a drop of leaf water potential that intensifies the change in transpiration and decreases WUE accordingly. However, our study found that ST had a significant positive effect on WUE (Figure 4), which may be supported by reduced stomatal conductance and high internal resistance of the thick epidermis (Latif et al., 2014). Our results also showed that ST affects plant WUE by affecting soil microbes [i.e., MBC (*P* < 0.05) and MBC:MBP] indirectly and positively. Soil microbes can form parasitic, symbiotic, or saprophytic relationships with plants directly or by promoting the decomposition of SOM, nutrient transformation, oxidation, nitration and the N biogeochemical processes of soil (van der Heijden et al., 2008). The rise of ST increased the activity of soil microbes, which affected the extracellular enzymes involved in SOM decomposition (Li et al., 2022), the regulation of nutrient cycling and thus affecting plant WUE.

### Effects of Soil Nutrients Conditions on Plant Water Use Efficiency

Soil moisture is one of the crucial factors affecting soil nutrient cycling both directly and indirectly. Plants consume excessive amounts of soil water content and nutrients during periods of rapid growth and reproduction, leading to plant growth being gradually limited by N and P (Deng et al., 2021). Our results showed that altered precipitation had a significant effect on TN and TN:TP positively at the initial growth stage (Table 3), because SM accelerates the decomposition of SOM with an increase in precipitation, and leading to the accumulation of soil TN (Cui et al., 2019; Deng et al., 2021). In our study, there was a significant negative correlation between WUE and soil TN of *A. gmelinii* (Figure 4). More C is often fixed by plants in low-N soil and more biomass per unit of N is produced (high N use efficiency, NUE) (Aerts and Chapin, 2000). Some studies have shown that there is a trade-off between plant WUE and NUE, where plants often decrease their WUE with the increase in NUE (Gong et al., 2011). Physiological constraints in the leaf have been used to explain the trade-off when plants increase intercellular CO_2_ concentration of leaves by opening their stomata, which increases photosynthesis per unit of N in leaves. However, it will increase transpiration and reducing WUE (Gong et al., 2011). Therefore, reduced WUE by greater water availability frequently increases NUE. Similarly, N in leaf tissue increases when N availability increases (NUE decreases), which promotes the photosynthetic capacity of leaves, thereby increasing WUE (Ripullone et al., 2004).

**TABLE 3:** Response of soil physical-chemical properties to precipitation manipulation at different growth stages.

Growing stages	Treatments	SM (%)	ST (°C)	SOC (g/kg)	TN (g/kg)	TP (g/kg)	SOC:TN	SOC:TP	TN:TP	NH_4_^+^–N (mg/kg)	NO_3_^–^–N (mg/kg)	AN (mg/kg)	AP (mg/kg)
Initial stage	CK	8.76 ± 1.57Bb	21.5 ± 2.07Cb	20.56 ± 0.8Ba	2.23 ± 0.10Aab	1.42 ± 0.03Aa	9.24 ± 0.52Ba	14.51 ± 0.56Ba	1.57 ± 0.07ABab	11.65 ± 2.13Aa	4.29 ± 1.07Bb	144.55 ± 11.32Aa	2.66 ± 0.33Bb
	DP	5.4 ± 1.6Bc	25.4 ± 5.11Ba	20.36 ± 1.09Ba	2.15 ± 0.08Ab	1.41 ± 0.06Aa	9.46 ± 0.45Ba	14.44 ± 0.86Ba	1.53 ± 0.07Ab	10.78 ± 2.17Aa	3.51 ± 0.85Bb	152.25 ± 3.91Aa	2.63 ± 0.46Bb
	IP	10.75 ± 1.34Ba	23.0 ± 3.32Cb	21.01 ± 1.85Aa	2.30 ± 0.11Aa	1.42 ± 0.02Aa	9.11 ± 0.55Ba	14.86 ± 1.28Aa	1.63 ± 0.07Aa	11.28 ± 2.00ABa	5.45 ± 1.53Ba	158.11 ± 8.73Aa	5.39 ± 1.51Aa
Middle stage	CK	12.68 ± 2.24Aa	27.6 ± 3.03Ba	20.09 ± 0.97Ba	2.27 ± 0.34Aa	1.37 ± 0.04Ba	8.96 ± 0.93Ba	14.65 ± 0.80Ba	1.66 ± 0.25Aa	9.52 ± 1.63Ba	5.75 ± 0.58Ab	160.13 ± 4.71Aa	4.42 ± 0.67Aa
	DP	10.44 ± 2.74Ab	27.4 ± 2.64Ba	19.97 ± 0.76Ba	2.15 ± 0.09Aa	1.46 ± 0.17Aa	9.30 ± 0.30Ba	13.90 ± 1.62Ba	1.50 ± 0.17Aa	9.10 ± 1.45Aa	11.18 ± 2.01Aa	155.40 ± 9.45Aa	4.18 ± 0.93Aa
	IP	14.22 ± 4.4Aa	26.7 ± 2.27Ba	20.68 ± 1.33Aa	2.23 ± 0.11Aa	1.40 ± 0.12Aa	9.28 ± 0.44Ba	14.88 ± 1.19Aa	1.61 ± 0.15Aa	9.52 ± 0.97Ba	10.06 ± 1.07Aa	159.83 ± 8.25Aa	3.70 ± 0.92Ba
Final stage	CK	6.86 ± 1.37Cb	29.4 ± 2.14Ab	22.19 ± 1.12Aa	2.09 ± 0.09Aa	1.40 ± 0.05ABa	10.62 ± 0.51Aa	15.88 ± 1.10Aa	1.49 ± 0.07Ba	11.19 ± 2.01ABab	5.28 ± 0.92Ab	151.42 ± 8.24Aa	3.81 ± 0.91Bab
	DP	2.89 ± 0.94Cc	31.9 ± 3.13Aa	21.80 ± 1.08Aa	2.10 ± 0.12Aa	1.40 ± 0.06Aa	10.40 ± 0.37Aa	15.59 ± 0.70Aa	1.50 ± 0.08Aa	9.82 ± 1.15Ab	3.51 ± 0.61Bc	144.90 ± 14.27Aa	3.49 ± 0.56Bb
	IP	8.66 ± 1.26Ca	28.5 ± 0.99Ab	22.22 ± 1.88Aa	2.16 ± 0.25Aa	1.42 ± 0.07Aa	10.34 ± 0.76Aa	15.67 ± 1.27Aa	1.52 ± 0.16Aa	12.62 ± 2.56Aa	8.47 ± 2.86Aa	155.75 ± 9.42Aa	4.51 ± 0.75ABa

*CK, ambient precipitation; DP, decrease precipitation (50%); IP, increase precipitation (50%); SM, soil moisture; ST, soil temperature; SOC, soil organic carbon; TN, soil total nitrogen; TP, soil total phosphorus; SOC:TN, SOC:TN ratio; SOC:TP, SOC:TP ratio; TN:TP, TN:TP ratio; NH_4_^+^–N, soil NH_4_^+^–N content; NO_3_^–^–N, soil NO_3_^–^–N content; AN, soil available nitrogen content; AP, soil available phosphorus content. Different small letters mean significant difference between different treatments at the 0.05 level; different capital letters mean significant difference between different growing stages at the 0.05 level.*

Soil inorganic N mainly resulted from the ammonification and nitrification activities of soil microorganisms, which transform soil organic N into NH_4_^+^–N and NO_3_^–^–N (Chapin et al., 2011). In our study, the NH_4_^+^–N and NO_3_^–^–N showed a decreasing trend under DP conditions (Table 3), which may be related to the reduction in ammonification and nitrification rate. In addition, NH_4_^+^–N played a negatively critical role in WUE, but NO_3_^–^–N had a significant positive effect on WUE (Figure 4). Giese et al. (2011) observed that the pools of NO_3_^–^–N and NH_4_^+^–N were controlled by precipitation. When the precipitation is low, NH_4_^+^–N is the main form of inorganic N available, and the content of NO_3_^–^–N increases with an increase in precipitation. However, NH_4_^+^–N is an effective N that is converted from soil organic N by microbial mineralization, which can be absorbed by soil but not easily leached and can be directly absorbed and utilized by plants. Moreover, NO_3_^–^–N is negatively charged and is not easily absorbed by soil colloids, and as it is easily lost because of leaching with an increase in precipitation, it results in a large loss of N (Deluca et al., 2002). Soil NH_4_^+^–N and NO_3_^–^–N can be absorbed and utilized by plants and affecting the WUE directly.

Soil AP is an important indicator for evaluating the ability of soil to provide P. In our study, AP content increased with increasing precipitation (Table 3). This is because that increased precipitation can improve the utilization rate of P by plants, promote the growth of plant roots, enhances the osmotic regulation ability of plants, and maintain the balance between water absorption and water loss (Gutiérrez-Boem and Thomas, 1998). Moreover, increased precipitation will enhance soil microbial activity and thus accelerate the P cycle rate, which may lead to an increase in AP content. Our results also showed that soil AP had a significant positive effect on the WUE of *A. gmelinii*, and the reason is probably that AP can improve the water relationship of plants to a certain extent and significantly affect the growth status of plants. In addition, P plays a very important role in the improvement of photosynthesis as it is an important player in the photosynthetic process, and can affect the catalytic rate of Rubisco carboxylase either directly or indirectly, and promote photosynthesis in plants (Pieters et al., 2001).

### Effects of Soil Microbial Biomass Ecological Stoichiometry Characteristics on Plant Water Use Efficiency

**FIGURE 5:**
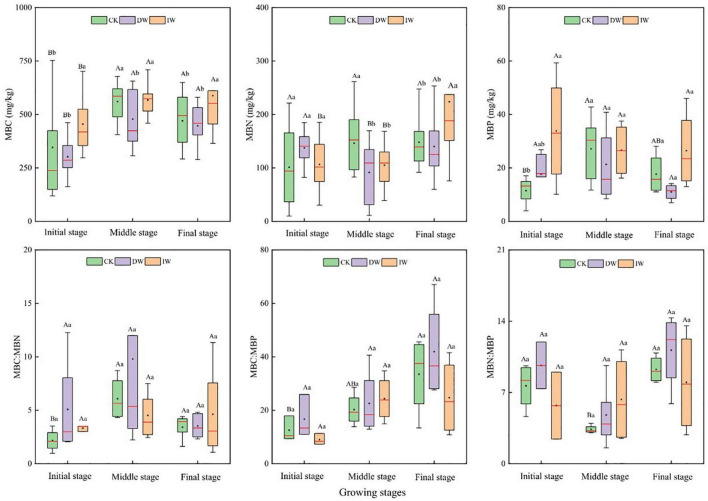
Response of soil microbial biomass C, N, P ecological stoichiometry characteristics to precipitation manipulation at different growth stages. CK, ambient precipitation; DP, decrease precipitation (–50%); IP, increase precipitation (+50%); MBC, microbial biomass carbon; MBN, microbial biomass nitrogen; MBP, microbial biomass phosphorus; MBC:MBN, MBC:MBN ratio; MBC:MBP, MBC:MBP ratio; MBN:MBP, MBN:MBP ratio. Different small letters mean significant difference among different treatments at the 0.05 level; different capital letters mean significant difference among different growing stages at the 0.05 level.

Changes in precipitation patterns may affect soil microbial biomass and its community structure by changing SM, ST, nutrient, and SOM input from aboveground and underground plant residues either directly or indirectly (Hawkes et al., 2011). Soil microbes are the driving force of SOM decomposition, which are closely related to C, N, and P cycling in soil (Xu et al., 2013). The ratios of microbe C:N:P can be used to determine the nutritional status and limitations of microbial growth (Zhou et al., 2019). In our study, soil microbial biomass was negatively affected by DP treatment generally (Figure 5), suggesting that the decrease in precipitation inhibited the proliferation and turnover of soil microorganisms (Wang et al., 2015). The consumer-driven nutrient cycling theory highlights that the biogeochemical cycle of nutrients can be promoted by microorganisms when these nutrients are required to meet their own needs (Elser and Urabe, 1999). Therefore, a high MBC represents high microbial activities, which reflects the strong ability of material circulation and promote plant growth and development of the ecosystem.

The MBC:MBP reflects the potential of soil microorganisms to regulate soil P availability (Li P. et al., 2019). A low MBC:MBP indicates that P is relatively abundant compared with soil SOC. At the time, microorganisms mineralize SOM to supplement the soil P pool, which further increases the restriction of soil C on microorganisms. A high MBC:MBP indicates that the soil is relatively rich in SOC and limited in P. At this time, microorganisms need to assimilate more P and maintain their normal growth and development, which shows that the ability of P fixation is improved. In our study, MBC and MBC:MBP affected WUE indirectly by affecting soil available nutrients (NH_4_^+^–N, NO_3_^–^–N, and AP). The higher MBC and the greater population of microorganisms influence the content of AN in soil through their N fixation and nitrification activities positively (Leff et al., 2015). Moreover, the release of extracellular enzymes and the decomposition of substrates by microorganisms also affect the supply of nutrients such as AP in the soil (Makino et al., 2010). However, a low MBC:MBP indicates that soil microorganisms have a greater potential to release P through circulation and thus play a significant role in replenishing the soil effective P pool.

## Conclusion

Changes in precipitation patterns had significant effects on the WUE of two functional groups in the grassland ecosystem. DP increased the WUE of grass species (*S. grandis* and *S. bungeana*) generally, but IP decreased WUE especially in forb species (*A. gmelinii*). The WUE was higher in grasses than forbs. Moreover, soil hydrothermal conditions, soil nutrients (i.e., NO_3_^–^–N, NH_4_^+^–N, and AP) and soil microbial activities were the main factors affecting the WUE of plants. Among them, ST played a key role. SM affected WUE through changes in ST, soil nutrients, and microbes indirectly. These results suggested that SM is not the main factor determining the change in WUE, but soil available nutrients play a critical role in affecting plant WUE in arid grassland ecosystems. In addition, the functional groups with high resource competitiveness and WUE such as grass species can be selected for vegetation restoration in arid and semi-arid areas. These findings provide a better understanding of the change and response process of plants WUE in grassland ecosystems to altered precipitation pattern, which will reveal the drivers and underlying mechanisms of WUE are crucial to predicting the impact of future climatic change on plant C and water cycling processes.

## Data Availability Statement

The raw data supporting the conclusions of this article will be made available by the authors, without undue reservation.

## Author Contributions

LDe, JPL, ZS, and ZH conceived the ideas and designed the study. XH, JWL, YL, LDo, XW, and WL measured soil nutrients and soil microbial biomass. XH wrote the first draft of the manuscript. All authors contributed critically to the drafts and gave final approval for publication.

## Conflict of Interest

The authors declare that the research was conducted in the absence of any commercial or financial relationships that could be construed as a potential conflict of interest.

## Publisher’s Note

All claims expressed in this article are solely those of the authors and do not necessarily represent those of their affiliated organizations, or those of the publisher, the editors and the reviewers. Any product that may be evaluated in this article, or claim that may be made by its manufacturer, is not guaranteed or endorsed by the publisher.
